# The Approved Societies and Their Medical Referees

**Published:** 1913-12-20

**Authors:** 


### THE PROFESSION OF MEDICINE.

(Contributions and Correspondence are Invited, that the Grave Questions of the Hour may be Examined from all Points of View.)

### THE APPROVED SOCIETIES AND THEIR MEDICAL REFEREES.

We have already on more than one occasion commented on the way in which the curse of party politics has damaged the Insurance Act in its scamped inception, in its inadequate discussion in Parliament, and in its subsequent administration as a device for the especial glorification of its author and the especial profit of the Ministerial party. With this end in view, the insured were refused (save in a handful of exceptional cases) that free choice of doctor which they had been promised; and many similar manœuvres in the proceedings of Insurance Committees during the last twelve months have been similarly inspired.

An excellent example of the way in which the panels and everything else connected with the Act are made to serve the turn of the politician is furnished by a letter to the *Daily News* of December 13. The writer is apparently an active member of the local Radical organisation in Hackney, and he beseeches all insured persons having grievances against their approved societies or their panel doctors to communicate with the local Liberal Association; and further suggests that in every borough the Liberal Association should start a committee to deal with such cases. Now this is in itself sufficiently humorous, coming from the source it does, and sufficiently improper in any case. But the train of reasoning upon which it is based is so extraordinary, and shows such a complete misconception of facts about the panels and the insured, that it is worth while shortly to recapitulate it.

The approved societies are accused of harshness by this critic, simply on the ground that they seek to protect their funds from misappropriation by malingerers. They are blamed for seeking the services of medical referees to detect and eliminate unfair demands for sickness benefit. By inference it is suggested that the panel doctors are more fit to decide whether an individual is or is not capable of working than the expert referees—an error so glaring and so entirely opposed to facts that it is probably useless to argue the point with anyone who seriously entertains it. It is further asserted that conflicts of opinion between "panellers" and referees are taking place "all over the country"; and all but stated that the latter are paid by the approved societies to certify patients as fit for work when they are in fact not fit.

Of course anyone who knows anything at all about the working of the Act knows that these grotesque assertions are quite unjustified. But this is the sort of stuff which passes for gospel in Ministerial party papers, and it throws a light upon the whole attitude of that party to the Act. By hook or crook the Act, the panels, the societies, and everything connected therewith, must serve a party turn somehow. A nice reflection for the panel men, surely, and a thorough justification of those who have refused to have anything to do with panels and to be dragged in triumph behind the chariots of politicians!

### Chemists and the Drug Fund.

From many parts of the country have come bitter complaints from the chemists who make up the prescriptions of the panel doctors concerning the inadequacy of their remuneration. It will be remembered that the Act provides one shilling and sixpence per head of the insured for a drug fund, with a further sixpence per head which goes to the chemists if required, but to the doctors if not required. As a matter of fact the full two shillings per head for drugs is found everywhere to be not nearly enough; and almost in every area the chemists have had to submit to their accounts being heavily cut down before they could be met out of the funds available. Not unnaturally, the chemists are displeased about this, and their grievance is a very real one. They themselves have no say in the selection of the drugs ordered, they merely dispense those prescribed by the paneller, who may not, and usually does not, consider for a moment the expense of what he orders. The tariff of prices which the chemist is allowed to charge is already so low that it barely pays him to undertake the work at all; and yet his bills after that are taxed down 25 or 30 or 40 per cent.

According to the *Manchester Guardian* a new system is being advocated at Salford, though this organ of Ministerial opinion adds the belief that there is not the faintest chance of the scheme being sanctioned by those in authority, the Insurance Commissioners. Briefly, it is proposed to make the doctor realise the financial aspect of prescription writing by paying him the two shillings which now goes to the drug fund, and then striking off it the cost of all his prescriptions. This ingenious idea would ensure a reasonable reward to the chemist for his work, and would impress the doctor with the need for economy in drugging. It may be added in parenthesis that the Salford chemists were being so sweated that they threatened to abandon panel work altogether next year unless something were done to meet their grievance. As we have said, the *Manchester Guardian* is not sanguine that the solution proposed in Salford will be permitted. But obviously something has got to be done, or the chemists will be giving up the Act all over the country.

This particular injustice under the Act has long been voiced in the pharmaceutical press; but so far it seems to have attracted comparatively little attention among the general public. It is, however, obviously a very serious matter.

